# Bis[μ_2_-bis­(diphenyl­phosphan­yl)methane-κ^2^ *P*:*P*′]bis­(μ_4_-diphenyl­phosphinato-κ^4^ *O*:*O*:*O*′:*O*′)bis­[μ_2_-trifluoro­methane­sulfonato­(0.546/0.454)]-κ^2^ *O*:*O*′;κ^2^ *O*:*O*-tetra­silver(I) acetonitrile disolvate

**DOI:** 10.1107/S160053681102931X

**Published:** 2011-07-23

**Authors:** Li-ping Tang, Chen Jia, Li-li Huang, Jia-Li Xie

**Affiliations:** a College of Chemistry, Sichuan University, Chengdu 610064, People’s Republic of China; b Sichuan College of Chemical Technology, Luzhou 646005, People’s Republic of China; c School of Chemistry and Chemical Engineering, Guangxi Normal University, Guilin 541004, People’s Republic of China

## Abstract

In the centrosymmetric tetra­nuclear title compound, [Ag_4_(C_12_H_10_O_2_P)_2_(CF_3_O_3_S)_2_(C_25_H_22_P_2_)_2_]·2CH_3_CN, the Ag^I^ atom is coordinated by one P atom from a bis­(diphenyl­phosphan­yl)methane (dppm) ligand, two O atoms from two diphenyl­phosphinate (dpp) ligands and one O atom from a trifluoro­methane­sulfonate (OTf) anion in a highly distorted tetra­hedral geometry. Four Ag^I^ atoms are bridged by two dppm ligands, two dpp ligands and two OTf anions, forming a tetra­nuclear complex. An weak intra­molecular Ag⋯Ag [3.2692 (14) Å] inter­action is observed. The OTf anion and one of the phenyl groups in the dppm ligand are disordered over two sets of positions in a 0.546 (4):0.454 (4) ratio. The 0.546-occupied OTf is bonded to two Ag atoms in a μ-(κ^2^ *O*:*O*′) mode, while the 0.454-occupied OTf is bonded in a μ-(κ^2^ *O*:*O*) mode. The methyl group of the acetonitrile solvent mol­ecule is also disordered over two positions with equal occupancy factors.

## Related literature

For related structures, see: Fournier *et al.* (2004 ▶); Matsumoto *et al.* (2001 ▶); Sun *et al.* (2011 ▶); Wei *et al.* (2004 ▶).
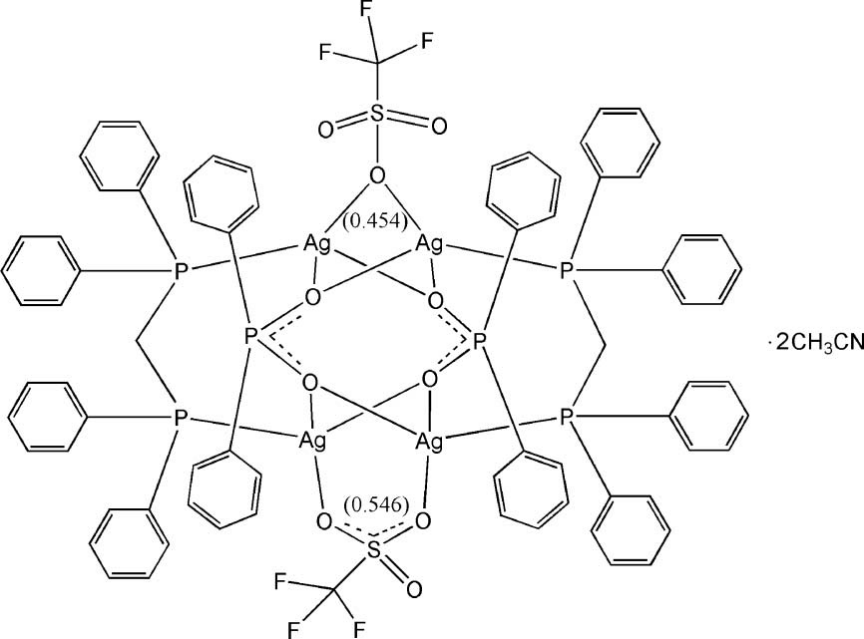


## Experimental

### 

#### Crystal data

[Ag_4_(C_12_H_10_O_2_P)_2_(CF_3_O_3_S)_2_(C_25_H_22_P_2_)_2_]·2C_2_H_3_N
*M* *_r_* = 2014.80
Monoclinic, 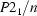

*a* = 11.730 (7) Å
*b* = 15.169 (9) Å
*c* = 23.688 (12) Å
β = 96.967 (7)°
*V* = 4184 (4) Å^3^
*Z* = 2
Mo *K*α radiation
μ = 1.16 mm^−1^
*T* = 296 K
0.23 × 0.19 × 0.16 mm

#### Data collection

Rigaku Mercury CCD diffractometer
Absorption correction: multi-scan (*CrystalClear*; Rigaku, 2005 ▶) *T*_min_ = 0.777, *T*_max_ = 0.837
41763 measured reflections
9645 independent reflections
7705 reflections with *I* > 2σ(*I*)
*R*_int_ = 0.055

#### Refinement

*R*[*F* ^2^ > 2σ(*F* ^2^)] = 0.055
*wR*(*F* ^2^) = 0.144
*S* = 1.01
9645 reflections
610 parameters
1173 restraints
H-atom parameters constrained
Δρ_max_ = 0.64 e Å^−3^
Δρ_min_ = −0.61 e Å^−3^

### 

Data collection: *CrystalClear* (Rigaku, 2005 ▶); cell refinement: *CrystalClear*; data reduction: *CrystalClear*; program(s) used to solve structure: *SHELXS97* (Sheldrick, 2008 ▶); program(s) used to refine structure: *SHELXL97* (Sheldrick, 2008 ▶); molecular graphics: *SHELXTL* (Sheldrick, 2008 ▶); software used to prepare material for publication: *SHELXTL*.

## Supplementary Material

Crystal structure: contains datablock(s) I, global. DOI: 10.1107/S160053681102931X/hy2447sup1.cif

Structure factors: contains datablock(s) I. DOI: 10.1107/S160053681102931X/hy2447Isup2.hkl

Additional supplementary materials:  crystallographic information; 3D view; checkCIF report

## Figures and Tables

**Table 1:** Selected bond lengths (Å)

Ag1—O1	2.270 (3)
Ag1—O2^i^	2.435 (3)
Ag1—O3	2.557 (8)
Ag1—O3*B*	2.672 (11)
Ag1—P2	2.3554 (15)
Ag2—O1^i^	2.451 (3)
Ag2—O2	2.280 (3)
Ag2—O5^i^	2.619 (8)
Ag2—O3*B*^i^	2.795 (10)
Ag2—P3	2.3676 (16)

Symmetry code: (i) 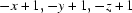.

**Fig. 1.**
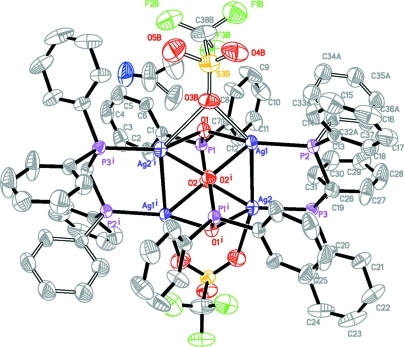
The molecular structure of the title complex. Displacement ellipsoids are drawn at the 50% probability level. The 0.454-occupied OTf anion and phenyl group are shown with open bonds. [Symmetry code: (i) 1-x, 1-y, 1-z.]

## supplementary crystallographic information

### Comment

Silver clusters have been intensively reported in many fields of chemistry (Fournier *et al.*, 2004; Wei *et al.*, 2004). Phosphine ligands, especially the type Ph_2_P(CH_2_)_n_PPh_2_ ligands, which are known to function as 'stabilizing units' on silver(I) centers, have been used in metal-rich silver chalcogenide clusters (Sun *et al.*, 2011). Information on the structures of tetranuclear silver(I) compounds by the reactions between silver(I) salt and bis(diphenylphosphine)methane (dppm) ligand continues to be reported (Matsumoto *et al.*, 2001).

In the title tetranuclear compound, the dppm ligand links two Ag^I^ atoms through the P atoms (Fig. 1). The Ag^I^ atom adopts a highly distorted tetrahedral geometry. The Ag—P bond distances are 2.3554 (15) and 2.3676 (16) Å and the Ag—O distances are in the range of 2.270 (3)–2.795 (10) Å (Table 1). An intramolecular Ag···Ag distance is 3.2692 (14) Å. The trifluoromethanesulfonate (OTf) anion and one of the phenyl groups in the dppm ligand are disordered over two sets of positions in an occupancy ratio of 0.546 (4):0.454 (4). The 0.546-occupied OTf is bonded to two Ag atoms in a µ-(κ^2^O:O') mode, while the 0.454-occupied OTf in a µ-(κ^2^O:O) mode (Fig. 1).

### Experimental

Silver trifluoromethanesulfonate (0.052 g, 0.2 mmol) was added with stirring to a solution of diphenylphosphinic acid (0.022 g, 0.1 mmol) and bis(diphenylphosphine)methane (0.038 g, 0.1 mmol) in CH_3_CN (5 ml). The resulting colorless solution was allowed to stir for 1 h. By slow diffusion of diethyl ether into the solution, prismatic colorless crystals were formed suitable for X-ray diffraction analysis (yield: 20%).

### Refinement

H atoms were positioned geometrically and refined as riding atoms, with C—H = 0.93 (CH), 0.97 (CH_2_) and 0.96 (CH_3_) Å and with *U*_iso_(H) = 1.2(1.5 for methyl)*U*_eq_(C).

### Crystal data

**Table:**

[Ag_4_(C_12_H_10_O_2_P)_2_(CF_3_O_3_S)_2_(C_25_H_22_P_2_)_2_]·2C_2_H_3_N	*F*(000) = 2016
*M**_r_* = 2014.80	*D*_x_ = 1.599 Mg m^−^^3^
Monoclinic, *P*2_1_/*n*	Mo *K*α radiation, λ = 0.71073 Å
Hall symbol: -P 2yn	Cell parameters from 9952 reflections
*a* = 11.730 (7) Å	θ = 1.6–27.5°
*b* = 15.169 (9) Å	µ = 1.16 mm^−^^1^
*c* = 23.688 (12) Å	*T* = 296 K
β = 96.967 (7)°	Prism, colorless
*V* = 4184 (4) Å^3^	0.23 × 0.19 × 0.16 mm
*Z* = 2	

### Data collection

**Table:**

Rigaku Mercury CCD diffractometer	9645 independent reflections
Radiation source: fine-focus sealed tube	7705 reflections with *I* > 2σ(*I*)
graphite	*R*_int_ = 0.055
Detector resolution: 13.6612 pixels mm^-1^	θ_max_ = 27.6°, θ_min_ = 2.5°
ω scans	*h* = −15→15
Absorption correction: multi-scan (*CrystalClear*; Rigaku, 2005)	*k* = −19→19
*T*_min_ = 0.777, *T*_max_ = 0.837	*l* = −30→30
41763 measured reflections	

### Refinement

**Table:**

Refinement on *F*^2^	Primary atom site location: structure-invariant direct methods
Least-squares matrix: full	Secondary atom site location: difference Fourier map
*R*[*F*^2^ > 2σ(*F*^2^)] = 0.055	Hydrogen site location: inferred from neighbouring sites
*wR*(*F*^2^) = 0.144	H-atom parameters constrained
*S* = 1.01	*w* = 1/[σ^2^(*F*_o_^2^) + (0.0595*P*)^2^ + 6.765*P*] where *P* = (*F*_o_^2^ + 2*F*_c_^2^)/3
9645 reflections	(Δ/σ)_max_ = 0.001
610 parameters	Δρ_max_ = 0.64 e Å^−^^3^
1173 restraints	Δρ_min_ = −0.61 e Å^−^^3^

### Fractional atomic coordinates and isotropic or equivalent isotropic displacement parameters (Å^2^)

**Table:**

	*x*	*y*	*z*	*U*_iso_*/*U*_eq_	Occ. (<1)
Ag1	0.47428 (3)	0.41739 (2)	0.415893 (15)	0.05183 (11)	
Ag2	0.33273 (3)	0.42158 (2)	0.526552 (16)	0.05416 (12)	
P1	0.37541 (9)	0.60694 (7)	0.45735 (5)	0.0427 (2)	
P2	0.37149 (10)	0.28791 (7)	0.38828 (5)	0.0456 (2)	
P3	0.24066 (9)	0.28840 (7)	0.49519 (5)	0.0461 (2)	
O1	0.4690 (2)	0.5658 (2)	0.42767 (13)	0.0487 (7)	
O2	0.3558 (3)	0.56941 (19)	0.51457 (13)	0.0501 (7)	
C1	0.4054 (4)	0.7227 (3)	0.46521 (19)	0.0502 (10)	
C2	0.3628 (5)	0.7712 (4)	0.5078 (3)	0.0739 (16)	
H2A	0.3261	0.7437	0.5357	0.089*	
C3	0.3774 (6)	0.8633 (5)	0.5072 (4)	0.102 (3)	
H3A	0.3496	0.8973	0.5352	0.122*	
C4	0.4314 (8)	0.9038 (5)	0.4663 (5)	0.115 (3)	
H4A	0.4380	0.9648	0.4659	0.138*	
C5	0.4748 (7)	0.8553 (5)	0.4268 (4)	0.105 (3)	
H5A	0.5139	0.8835	0.4000	0.126*	
C6	0.4632 (5)	0.7655 (4)	0.4246 (2)	0.0687 (14)	
H6A	0.4934	0.7332	0.3965	0.082*	
C7	0.2421 (4)	0.6076 (3)	0.41141 (19)	0.0499 (10)	
C8	0.2426 (6)	0.6155 (5)	0.3537 (2)	0.093 (2)	
H8A	0.3109	0.6132	0.3375	0.111*	
C9	0.1359 (7)	0.6272 (7)	0.3197 (3)	0.127 (3)	
H9A	0.1348	0.6358	0.2807	0.153*	
C10	0.0358 (6)	0.6261 (6)	0.3424 (4)	0.109 (3)	
H10A	−0.0337	0.6299	0.3190	0.131*	
C11	0.0372 (5)	0.6195 (5)	0.3986 (3)	0.091 (2)	
H11A	−0.0316	0.6204	0.4143	0.109*	
C12	0.1395 (4)	0.6114 (4)	0.4336 (3)	0.0700 (14)	
H12A	0.1389	0.6085	0.4728	0.084*	
C13	0.4580 (4)	0.2064 (3)	0.35683 (19)	0.0542 (11)	
C14	0.5547 (5)	0.2354 (5)	0.3329 (3)	0.0808 (17)	
H14A	0.5737	0.2949	0.3343	0.097*	
C15	0.6219 (6)	0.1770 (7)	0.3075 (3)	0.103 (2)	
H15A	0.6856	0.1973	0.2916	0.124*	
C16	0.5964 (8)	0.0908 (6)	0.3056 (3)	0.106 (3)	
H16A	0.6424	0.0519	0.2882	0.127*	
C17	0.5038 (8)	0.0600 (4)	0.3288 (3)	0.100 (3)	
H17A	0.4877	−0.0001	0.3277	0.121*	
C18	0.4324 (6)	0.1178 (4)	0.3544 (2)	0.0771 (16)	
H18A	0.3683	0.0965	0.3696	0.093*	
C19	0.3169 (4)	0.2252 (3)	0.44563 (18)	0.0484 (9)	
H19A	0.2655	0.1799	0.4285	0.058*	
H19B	0.3813	0.1956	0.4673	0.058*	
C20	0.2295 (4)	0.2085 (3)	0.5520 (2)	0.0575 (11)	
C21	0.2167 (6)	0.1196 (4)	0.5430 (3)	0.0844 (18)	
H21A	0.2140	0.0969	0.5064	0.101*	
C22	0.2077 (7)	0.0631 (5)	0.5885 (4)	0.111 (3)	
H22A	0.1982	0.0029	0.5820	0.134*	
C23	0.2127 (8)	0.0948 (6)	0.6421 (4)	0.125 (4)	
H23A	0.2080	0.0566	0.6725	0.150*	
C24	0.2245 (7)	0.1826 (7)	0.6513 (3)	0.109 (3)	
H24A	0.2267	0.2043	0.6881	0.130*	
C25	0.2334 (5)	0.2407 (5)	0.6069 (2)	0.0773 (16)	
H25A	0.2418	0.3009	0.6139	0.093*	
C26	0.0955 (4)	0.2986 (3)	0.4593 (2)	0.0548 (11)	
C27	0.0297 (6)	0.2276 (5)	0.4417 (4)	0.122 (3)	
H27A	0.0580	0.1710	0.4492	0.146*	
C28	−0.0801 (6)	0.2391 (6)	0.4125 (5)	0.135 (4)	
H28A	−0.1246	0.1900	0.4013	0.162*	
C29	−0.1217 (5)	0.3196 (6)	0.4006 (3)	0.094 (2)	
H29A	−0.1938	0.3267	0.3800	0.112*	
C30	−0.0595 (6)	0.3893 (5)	0.4184 (4)	0.107 (3)	
H30A	−0.0897	0.4453	0.4110	0.128*	
C31	0.0496 (5)	0.3807 (4)	0.4478 (3)	0.0821 (18)	
H31A	0.0917	0.4307	0.4597	0.099*	
C32	0.2483 (6)	0.3084 (5)	0.3333 (3)	0.062 (2)	0.546 (4)
C33	0.1831 (7)	0.3845 (5)	0.3358 (3)	0.083 (3)	0.546 (4)
H33A	0.2057	0.4275	0.3628	0.100*	0.546 (4)
C34	0.0843 (7)	0.3964 (5)	0.2978 (4)	0.092 (3)	0.546 (4)
H34A	0.0407	0.4473	0.2994	0.111*	0.546 (4)
C35	0.0506 (7)	0.3321 (6)	0.2574 (4)	0.109 (3)	0.546 (4)
H35A	−0.0155	0.3400	0.2320	0.131*	0.546 (4)
C36	0.1158 (8)	0.2560 (6)	0.2549 (4)	0.107 (3)	0.546 (4)
H36A	0.0932	0.2130	0.2278	0.129*	0.546 (4)
C37	0.2146 (8)	0.2441 (5)	0.2928 (4)	0.102 (3)	0.546 (4)
H37A	0.2582	0.1932	0.2912	0.122*	0.546 (4)
C32A	0.2485 (8)	0.3032 (7)	0.3374 (4)	0.069 (3)	0.454 (4)
C33A	0.2426 (9)	0.3856 (7)	0.3116 (5)	0.106 (3)	0.454 (4)
H33B	0.3025	0.4256	0.3196	0.127*	0.454 (4)
C34A	0.1473 (11)	0.4083 (6)	0.2737 (5)	0.109 (3)	0.454 (4)
H34B	0.1433	0.4634	0.2564	0.131*	0.454 (4)
C35A	0.0578 (9)	0.3486 (7)	0.2616 (5)	0.109 (3)	0.454 (4)
H35B	−0.0061	0.3637	0.2363	0.131*	0.454 (4)
C36A	0.0636 (8)	0.2661 (7)	0.2875 (5)	0.095 (3)	0.454 (4)
H36B	0.0037	0.2262	0.2794	0.114*	0.454 (4)
C37A	0.1590 (9)	0.2435 (6)	0.3254 (4)	0.087 (3)	0.454 (4)
H37B	0.1629	0.1883	0.3426	0.104*	0.454 (4)
S3	0.6892 (4)	0.4984 (3)	0.3233 (2)	0.0534 (9)	0.546 (4)
F1	0.5949 (11)	0.5743 (8)	0.2281 (4)	0.129 (3)	0.546 (4)
F2	0.4775 (11)	0.5059 (10)	0.2761 (5)	0.171 (4)	0.546 (4)
F3	0.5531 (10)	0.6323 (7)	0.3112 (4)	0.116 (3)	0.546 (4)
O3	0.6061 (8)	0.4384 (7)	0.3385 (4)	0.105 (2)	0.546 (4)
O4	0.7736 (8)	0.4696 (7)	0.2896 (4)	0.089 (2)	0.546 (4)
O5	0.7325 (7)	0.5485 (6)	0.3733 (3)	0.090 (2)	0.546 (4)
C38	0.5805 (10)	0.5538 (7)	0.2844 (4)	0.112 (3)	0.546 (4)
S3B	0.6771 (9)	0.5015 (6)	0.3148 (4)	0.101 (3)	0.454 (4)
F1B	0.5476 (12)	0.5333 (11)	0.2273 (6)	0.128 (4)	0.454 (4)
F2B	0.6761 (10)	0.6489 (7)	0.2723 (5)	0.131 (3)	0.454 (4)
F3B	0.4952 (11)	0.5877 (11)	0.2957 (6)	0.128 (4)	0.454 (4)
O3B	0.6535 (9)	0.4851 (8)	0.3711 (4)	0.085 (3)	0.454 (4)
O4B	0.7099 (14)	0.4260 (9)	0.2841 (5)	0.113 (3)	0.454 (4)
O5B	0.7712 (12)	0.5619 (10)	0.3130 (6)	0.139 (4)	0.454 (4)
C38B	0.6009 (10)	0.5791 (9)	0.2748 (6)	0.112 (4)	0.454 (4)
N1	0.1746 (8)	1.0190 (5)	0.4011 (4)	0.148 (3)	
C39	0.1507 (8)	0.9478 (8)	0.3896 (6)	0.163 (5)	
C40A	0.150 (2)	0.8812 (15)	0.3496 (14)	0.212 (9)	0.50
H40A	0.2094	0.8395	0.3615	0.318*	0.50
H40B	0.1624	0.9060	0.3135	0.318*	0.50
H40C	0.0767	0.8518	0.3459	0.318*	0.50
C40B	0.1071 (16)	0.8694 (11)	0.4110 (12)	0.162 (7)	0.50
H40D	0.0406	0.8506	0.3863	0.243*	0.50
H40E	0.0863	0.8799	0.4484	0.243*	0.50
H40F	0.1649	0.8243	0.4129	0.243*	0.50

### Atomic displacement parameters (Å^2^)

**Table:**

	*U*^11^	*U*^22^	*U*^33^	*U*^12^	*U*^13^	*U*^23^
Ag1	0.0551 (2)	0.0487 (2)	0.0512 (2)	−0.00963 (15)	0.00500 (15)	−0.00754 (14)
Ag2	0.0505 (2)	0.0516 (2)	0.0602 (2)	−0.00592 (15)	0.00608 (15)	−0.00487 (15)
P1	0.0388 (5)	0.0407 (5)	0.0469 (6)	0.0029 (4)	−0.0016 (4)	−0.0006 (4)
P2	0.0493 (6)	0.0425 (6)	0.0446 (6)	−0.0038 (5)	0.0038 (4)	−0.0052 (4)
P3	0.0407 (5)	0.0460 (6)	0.0517 (6)	−0.0020 (4)	0.0063 (4)	0.0041 (5)
O1	0.0421 (15)	0.0478 (16)	0.0559 (17)	0.0036 (12)	0.0049 (12)	−0.0051 (13)
O2	0.0537 (17)	0.0481 (17)	0.0466 (16)	0.0017 (13)	−0.0021 (13)	0.0021 (12)
C1	0.045 (2)	0.041 (2)	0.061 (3)	0.0041 (18)	−0.0083 (19)	−0.0002 (18)
C2	0.067 (3)	0.056 (3)	0.094 (4)	0.012 (3)	−0.009 (3)	−0.024 (3)
C3	0.086 (5)	0.067 (4)	0.142 (7)	0.027 (4)	−0.027 (4)	−0.042 (4)
C4	0.109 (6)	0.043 (3)	0.176 (9)	−0.007 (4)	−0.055 (6)	0.008 (4)
C5	0.115 (6)	0.059 (4)	0.130 (6)	−0.018 (4)	−0.031 (5)	0.036 (4)
C6	0.073 (3)	0.057 (3)	0.072 (3)	−0.009 (3)	−0.008 (3)	0.020 (2)
C7	0.045 (2)	0.049 (2)	0.053 (2)	0.0040 (19)	−0.0026 (18)	−0.0054 (19)
C8	0.076 (4)	0.146 (6)	0.053 (3)	0.007 (4)	−0.006 (3)	−0.010 (4)
C9	0.097 (6)	0.210 (10)	0.064 (4)	0.013 (6)	−0.031 (4)	−0.004 (5)
C10	0.067 (4)	0.147 (7)	0.103 (6)	0.017 (4)	−0.036 (4)	−0.010 (5)
C11	0.045 (3)	0.107 (5)	0.115 (6)	0.011 (3)	−0.011 (3)	−0.010 (4)
C12	0.052 (3)	0.076 (4)	0.078 (4)	0.011 (3)	−0.006 (2)	−0.009 (3)
C13	0.062 (3)	0.054 (3)	0.047 (2)	0.006 (2)	0.0042 (19)	−0.0079 (19)
C14	0.070 (4)	0.091 (4)	0.086 (4)	0.001 (3)	0.025 (3)	−0.023 (3)
C15	0.085 (5)	0.140 (7)	0.089 (5)	0.027 (5)	0.031 (4)	−0.015 (5)
C16	0.130 (7)	0.121 (7)	0.066 (4)	0.071 (6)	0.015 (4)	−0.004 (4)
C17	0.176 (8)	0.062 (4)	0.063 (4)	0.039 (4)	0.014 (4)	−0.003 (3)
C18	0.117 (5)	0.053 (3)	0.065 (3)	0.009 (3)	0.025 (3)	−0.005 (2)
C19	0.044 (2)	0.044 (2)	0.057 (2)	−0.0055 (18)	0.0060 (18)	0.0000 (18)
C20	0.052 (3)	0.062 (3)	0.060 (3)	0.007 (2)	0.015 (2)	0.016 (2)
C21	0.099 (5)	0.069 (4)	0.091 (4)	0.006 (3)	0.039 (4)	0.024 (3)
C22	0.121 (6)	0.079 (5)	0.145 (7)	0.018 (4)	0.060 (5)	0.054 (5)
C23	0.133 (7)	0.130 (7)	0.125 (7)	0.052 (6)	0.066 (6)	0.081 (6)
C24	0.103 (5)	0.159 (8)	0.069 (4)	0.048 (5)	0.029 (4)	0.042 (5)
C25	0.074 (4)	0.101 (5)	0.058 (3)	0.014 (3)	0.016 (3)	0.022 (3)
C26	0.038 (2)	0.061 (3)	0.066 (3)	−0.006 (2)	0.0057 (19)	0.005 (2)
C27	0.061 (4)	0.062 (4)	0.227 (10)	−0.007 (3)	−0.045 (5)	0.003 (5)
C28	0.064 (4)	0.104 (6)	0.222 (10)	−0.020 (4)	−0.045 (5)	0.005 (6)
C29	0.050 (3)	0.119 (6)	0.107 (5)	−0.005 (4)	−0.011 (3)	0.027 (4)
C30	0.072 (4)	0.088 (5)	0.149 (7)	0.013 (4)	−0.034 (4)	0.025 (5)
C31	0.065 (3)	0.066 (4)	0.108 (5)	0.005 (3)	−0.019 (3)	0.013 (3)
C32	0.069 (5)	0.052 (4)	0.060 (4)	−0.010 (4)	−0.012 (4)	0.001 (4)
C33	0.089 (5)	0.072 (5)	0.079 (5)	0.002 (5)	−0.026 (4)	0.003 (4)
C34	0.092 (5)	0.085 (5)	0.091 (5)	0.004 (5)	−0.023 (5)	0.012 (5)
C35	0.106 (6)	0.094 (5)	0.112 (6)	−0.017 (5)	−0.046 (5)	0.010 (5)
C36	0.107 (6)	0.101 (5)	0.102 (6)	−0.009 (5)	−0.039 (5)	−0.014 (5)
C37	0.102 (6)	0.087 (5)	0.104 (6)	−0.007 (5)	−0.039 (5)	−0.008 (5)
C32A	0.075 (5)	0.060 (5)	0.067 (5)	−0.013 (5)	−0.014 (5)	0.007 (5)
C33A	0.109 (6)	0.089 (6)	0.106 (6)	−0.014 (5)	−0.040 (5)	0.023 (5)
C34A	0.108 (6)	0.096 (6)	0.111 (6)	−0.015 (5)	−0.036 (5)	0.019 (5)
C35A	0.107 (6)	0.096 (6)	0.111 (6)	−0.014 (5)	−0.044 (5)	0.017 (5)
C36A	0.091 (6)	0.090 (5)	0.095 (6)	−0.019 (5)	−0.029 (5)	−0.003 (5)
C37A	0.088 (6)	0.079 (5)	0.084 (6)	−0.019 (5)	−0.030 (5)	0.007 (5)
S3	0.0522 (16)	0.0584 (18)	0.0537 (18)	0.0065 (14)	0.0231 (14)	0.0032 (14)
F1	0.153 (8)	0.160 (8)	0.076 (5)	0.043 (6)	0.025 (5)	0.049 (5)
F2	0.154 (7)	0.197 (8)	0.151 (7)	−0.012 (7)	−0.027 (6)	0.005 (7)
F3	0.136 (7)	0.114 (6)	0.099 (5)	0.042 (5)	0.014 (5)	−0.004 (4)
O3	0.113 (5)	0.118 (6)	0.093 (5)	−0.016 (5)	0.049 (4)	0.005 (4)
O4	0.090 (5)	0.114 (6)	0.072 (4)	0.023 (5)	0.049 (4)	0.009 (4)
O5	0.100 (5)	0.099 (5)	0.073 (5)	0.000 (5)	0.022 (4)	−0.007 (4)
C38	0.105 (6)	0.138 (7)	0.094 (6)	0.051 (5)	0.015 (5)	0.040 (6)
S3B	0.114 (5)	0.113 (5)	0.078 (4)	−0.020 (4)	0.025 (3)	0.001 (3)
F1B	0.116 (8)	0.157 (9)	0.110 (7)	0.004 (7)	0.008 (6)	0.003 (7)
F2B	0.151 (7)	0.123 (7)	0.125 (6)	0.012 (6)	0.043 (6)	0.038 (5)
F3B	0.109 (7)	0.162 (9)	0.122 (7)	0.061 (6)	0.045 (6)	0.020 (7)
O3B	0.086 (6)	0.115 (7)	0.060 (5)	0.006 (5)	0.029 (4)	0.005 (5)
O4B	0.140 (8)	0.126 (8)	0.078 (6)	0.040 (7)	0.033 (6)	−0.011 (6)
O5B	0.134 (8)	0.156 (8)	0.133 (8)	−0.034 (7)	0.047 (7)	0.001 (7)
C38B	0.119 (7)	0.133 (7)	0.091 (7)	0.019 (7)	0.049 (6)	0.028 (7)
N1	0.151 (7)	0.080 (5)	0.205 (8)	−0.016 (5)	−0.011 (6)	−0.017 (5)
C39	0.092 (6)	0.102 (7)	0.276 (13)	0.005 (5)	−0.052 (7)	−0.025 (8)
C40A	0.176 (17)	0.173 (17)	0.27 (2)	0.027 (15)	−0.042 (17)	−0.088 (16)
C40B	0.092 (11)	0.120 (13)	0.261 (19)	−0.028 (10)	−0.032 (13)	0.057 (14)

### Geometric parameters (Å, °)

**Table:**

Ag1—O1	2.270 (3)	C22—H22A	0.9300
Ag1—O2^i^	2.435 (3)	C23—C24	1.353 (13)
Ag1—O3	2.557 (8)	C23—H23A	0.9300
Ag1—O3B	2.672 (11)	C24—C25	1.386 (9)
Ag1—P2	2.3554 (15)	C24—H24A	0.9300
Ag2—O1^i^	2.451 (3)	C25—H25A	0.9300
Ag2—O2	2.280 (3)	C26—C27	1.360 (8)
Ag2—O5^i^	2.619 (8)	C26—C31	1.371 (5)
Ag2—O3B^i^	2.795 (10)	C27—C28	1.396 (9)
Ag2—P3	2.3676 (16)	C27—H27A	0.9300
Ag1—Ag2	3.2692 (14)	C28—C29	1.333 (11)
P1—O1	1.509 (3)	C28—H28A	0.9300
P1—O2	1.513 (3)	C29—C30	1.324 (10)
P1—C7	1.794 (4)	C29—H29A	0.9300
P1—C1	1.796 (5)	C30—C31	1.387 (8)
P2—C32A	1.779 (7)	C30—H30A	0.9300
P2—C13	1.816 (5)	C31—H31A	0.9300
P2—C19	1.836 (4)	C32—C33	1.3900
P2—C32	1.851 (6)	C32—C37	1.3900
P3—C26	1.815 (5)	C33—C34	1.3900
P3—C20	1.827 (5)	C33—H33A	0.9300
P3—C19	1.831 (5)	C34—C35	1.3900
C1—C2	1.389 (7)	C34—H34A	0.9300
C1—C6	1.402 (7)	C35—C36	1.3900
C2—C3	1.409 (9)	C35—H35A	0.9300
C2—H2A	0.9300	C36—C37	1.3900
C3—C4	1.366 (12)	C36—H36A	0.9300
C3—H3A	0.9300	C37—H37A	0.9300
C4—C5	1.337 (12)	C32A—C33A	1.3900
C4—H4A	0.9300	C32A—C37A	1.3900
C5—C6	1.370 (9)	C33A—C34A	1.3900
C5—H5A	0.9300	C33A—H33B	0.9300
C6—H6A	0.9300	C34A—C35A	1.3900
C7—C12	1.371 (7)	C34A—H34B	0.9300
C7—C8	1.373 (7)	C35A—C36A	1.3900
C8—C9	1.415 (9)	C35A—H35B	0.9300
C8—H8A	0.9300	C36A—C37A	1.3900
C9—C10	1.350 (11)	C36A—H36B	0.9300
C9—H9A	0.9300	C37A—H37B	0.9300
C10—C11	1.332 (10)	S3—O3	1.412 (8)
C10—H10A	0.9300	S3—O4	1.415 (6)
C11—C12	1.379 (7)	S3—O5	1.446 (7)
C11—H11A	0.9300	S3—C38	1.702 (10)
C12—H12A	0.9300	F1—C38	1.399 (8)
C13—C18	1.376 (7)	F2—C38	1.402 (8)
C13—C14	1.399 (8)	F3—C38	1.405 (8)
C14—C15	1.372 (9)	S3B—O3B	1.416 (9)
C14—H14A	0.9300	S3B—O4B	1.434 (8)
C15—C16	1.340 (12)	S3B—O5B	1.439 (8)
C15—H15A	0.9300	S3B—C38B	1.696 (12)
C16—C17	1.359 (12)	F1B—C38B	1.403 (8)
C16—H16A	0.9300	F2B—C38B	1.384 (8)
C17—C18	1.401 (9)	F3B—C38B	1.396 (8)
C17—H17A	0.9300	N1—C39	1.141 (11)
C18—H18A	0.9300	C39—C40A	1.39 (2)
C19—H19A	0.9700	C39—C40B	1.413 (19)
C19—H19B	0.9700	C40A—H40A	0.9600
C20—C21	1.372 (8)	C40A—H40B	0.9600
C20—C25	1.385 (8)	C40A—H40C	0.9600
C21—C22	1.390 (9)	C40B—H40D	0.9600
C21—H21A	0.9300	C40B—H40E	0.9600
C22—C23	1.353 (13)	C40B—H40F	0.9600
			
O1—Ag1—P2	147.19 (8)	C20—C21—C22	120.2 (7)
O1—Ag1—O2^i^	82.38 (10)	C20—C21—H21A	119.9
P2—Ag1—O2^i^	127.29 (8)	C22—C21—H21A	119.9
O1—Ag1—O3	89.6 (2)	C23—C22—C21	120.6 (8)
P2—Ag1—O3	103.8 (2)	C23—C22—H22A	119.7
O2^i^—Ag1—O3	87.6 (3)	C21—C22—H22A	119.7
O1—Ag1—Ag2	81.85 (8)	C24—C23—C22	119.5 (7)
P2—Ag1—Ag2	86.92 (4)	C24—C23—H23A	120.2
O2^i^—Ag1—Ag2	84.82 (9)	C22—C23—H23A	120.2
O3—Ag1—Ag2	169.2 (2)	C23—C24—C25	121.3 (8)
O2—Ag2—P3	148.93 (8)	C23—C24—H24A	119.3
O2—Ag2—O1^i^	81.83 (10)	C25—C24—H24A	119.3
P3—Ag2—O1^i^	125.28 (8)	C20—C25—C24	119.4 (7)
O2—Ag2—Ag1	80.79 (8)	C20—C25—H25A	120.3
P3—Ag2—Ag1	89.34 (4)	C24—C25—H25A	120.3
O1^i^—Ag2—Ag1	79.01 (8)	C27—C26—C31	117.6 (5)
O1—P1—O2	117.39 (19)	C27—C26—P3	122.8 (4)
O1—P1—C7	110.5 (2)	C31—C26—P3	119.6 (4)
O2—P1—C7	109.2 (2)	C26—C27—C28	120.5 (7)
O1—P1—C1	107.9 (2)	C26—C27—H27A	119.7
O2—P1—C1	108.9 (2)	C28—C27—H27A	119.7
C7—P1—C1	101.7 (2)	C29—C28—C27	120.8 (7)
C32A—P2—C13	105.0 (4)	C29—C28—H28A	119.6
C32A—P2—C19	104.0 (4)	C27—C28—H28A	119.6
C13—P2—C19	102.3 (2)	C30—C29—C28	119.3 (6)
C13—P2—C32	104.6 (3)	C30—C29—H29A	120.3
C19—P2—C32	107.2 (3)	C28—C29—H29A	120.3
C32A—P2—Ag1	115.2 (3)	C29—C30—C31	121.7 (7)
C13—P2—Ag1	112.91 (17)	C29—C30—H30A	119.2
C19—P2—Ag1	116.07 (15)	C31—C30—H30A	119.2
C32—P2—Ag1	112.7 (3)	C26—C31—C30	120.1 (6)
C26—P3—C20	104.8 (2)	C26—C31—H31A	120.0
C26—P3—C19	104.3 (2)	C30—C31—H31A	120.0
C20—P3—C19	102.1 (2)	C33—C32—C37	120.0
C26—P3—Ag2	116.15 (16)	C33—C32—P2	119.7 (4)
C20—P3—Ag2	113.91 (18)	C37—C32—P2	120.0 (4)
C19—P3—Ag2	114.10 (14)	C32—C33—C34	120.0
P1—O1—Ag1	119.96 (17)	C32—C33—H33A	120.0
P1—O1—Ag2^i^	117.81 (16)	C34—C33—H33A	120.0
Ag1—O1—Ag2^i^	95.36 (10)	C33—C34—C35	120.0
P1—O2—Ag2	120.97 (17)	C33—C34—H34A	120.0
P1—O2—Ag1^i^	111.96 (17)	C35—C34—H34A	120.0
Ag2—O2—Ag1^i^	95.53 (10)	C36—C35—C34	120.0
C2—C1—C6	120.0 (5)	C36—C35—H35A	120.0
C2—C1—P1	120.6 (4)	C34—C35—H35A	120.0
C6—C1—P1	119.1 (4)	C35—C36—C37	120.0
C1—C2—C3	117.6 (7)	C35—C36—H36A	120.0
C1—C2—H2A	121.2	C37—C36—H36A	120.0
C3—C2—H2A	121.2	C36—C37—C32	120.0
C4—C3—C2	121.3 (7)	C36—C37—H37A	120.0
C4—C3—H3A	119.3	C32—C37—H37A	120.0
C2—C3—H3A	119.3	C33A—C32A—C37A	120.0
C5—C4—C3	119.9 (7)	C33A—C32A—P2	114.1 (6)
C5—C4—H4A	120.1	C37A—C32A—P2	125.8 (6)
C3—C4—H4A	120.1	C34A—C33A—C32A	120.0
C4—C5—C6	121.9 (8)	C34A—C33A—H33B	120.0
C4—C5—H5A	119.1	C32A—C33A—H33B	120.0
C6—C5—H5A	119.1	C33A—C34A—C35A	120.0
C5—C6—C1	119.2 (7)	C33A—C34A—H34B	120.0
C5—C6—H6A	120.4	C35A—C34A—H34B	120.0
C1—C6—H6A	120.4	C36A—C35A—C34A	120.0
C12—C7—C8	119.2 (5)	C36A—C35A—H35B	120.0
C12—C7—P1	120.6 (4)	C34A—C35A—H35B	120.0
C8—C7—P1	119.7 (4)	C37A—C36A—C35A	120.0
C7—C8—C9	117.9 (7)	C37A—C36A—H36B	120.0
C7—C8—H8A	121.1	C35A—C36A—H36B	120.0
C9—C8—H8A	121.1	C36A—C37A—C32A	120.0
C10—C9—C8	121.6 (7)	C36A—C37A—H37B	120.0
C10—C9—H9A	119.2	C32A—C37A—H37B	120.0
C8—C9—H9A	119.2	O3—S3—O4	119.8 (7)
C11—C10—C9	119.5 (6)	O3—S3—O5	108.4 (7)
C11—C10—H10A	120.2	O4—S3—O5	115.1 (7)
C9—C10—H10A	120.2	O3—S3—C38	87.8 (7)
C10—C11—C12	120.8 (7)	O4—S3—C38	112.1 (6)
C10—C11—H11A	119.6	O5—S3—C38	110.4 (6)
C12—C11—H11A	119.6	S3—O3—Ag1	139.4 (7)
C7—C12—C11	120.9 (6)	F1—C38—F2	100.5 (10)
C7—C12—H12A	119.6	F1—C38—F3	107.5 (10)
C11—C12—H12A	119.6	F2—C38—F3	105.2 (10)
C18—C13—C14	118.3 (5)	F1—C38—S3	117.2 (8)
C18—C13—P2	123.5 (4)	F2—C38—S3	113.3 (9)
C14—C13—P2	118.2 (4)	F3—C38—S3	111.8 (8)
C15—C14—C13	120.8 (7)	O3B—S3B—O4B	115.6 (10)
C15—C14—H14A	119.6	O3B—S3B—O5B	112.3 (10)
C13—C14—H14A	119.6	O4B—S3B—O5B	103.8 (11)
C16—C15—C14	120.4 (8)	O3B—S3B—C38B	119.9 (8)
C16—C15—H15A	119.8	O4B—S3B—C38B	115.2 (9)
C14—C15—H15A	119.8	O5B—S3B—C38B	84.0 (9)
C15—C16—C17	120.5 (7)	F2B—C38B—F3B	122.8 (14)
C15—C16—H16A	119.7	F2B—C38B—F1B	124.6 (13)
C17—C16—H16A	119.7	F3B—C38B—F1B	90.2 (12)
C16—C17—C18	120.6 (7)	F2B—C38B—S3B	105.0 (9)
C16—C17—H17A	119.7	F3B—C38B—S3B	107.3 (10)
C18—C17—H17A	119.7	F1B—C38B—S3B	104.9 (11)
C13—C18—C17	119.4 (7)	N1—C39—C40A	147 (2)
C13—C18—H18A	120.3	N1—C39—C40B	143 (2)
C17—C18—H18A	120.3	C40A—C39—C40B	70.0 (17)
P3—C19—P2	116.4 (2)	C39—C40A—H40A	109.5
P3—C19—H19A	108.2	C39—C40A—H40B	109.5
P2—C19—H19A	108.2	C39—C40A—H40C	109.5
P3—C19—H19B	108.2	C39—C40B—H40D	109.5
P2—C19—H19B	108.2	C39—C40B—H40E	109.5
H19A—C19—H19B	107.3	H40D—C40B—H40E	109.5
C21—C20—C25	118.9 (5)	C39—C40B—H40F	109.5
C21—C20—P3	123.7 (4)	H40D—C40B—H40F	109.5
C25—C20—P3	117.4 (4)	H40E—C40B—H40F	109.5
			
O1—Ag1—Ag2—O2	0.50 (10)	P2—C13—C14—C15	−178.7 (5)
P2—Ag1—Ag2—O2	−148.59 (8)	C13—C14—C15—C16	−0.5 (11)
O2^i^—Ag1—Ag2—O2	83.53 (11)	C14—C15—C16—C17	−0.2 (12)
O3—Ag1—Ag2—O2	38.0 (13)	C15—C16—C17—C18	1.0 (12)
O1—Ag1—Ag2—P3	150.92 (8)	C14—C13—C18—C17	0.6 (9)
P2—Ag1—Ag2—P3	1.83 (4)	P2—C13—C18—C17	179.4 (5)
O2^i^—Ag1—Ag2—P3	−126.06 (8)	C16—C17—C18—C13	−1.2 (10)
O3—Ag1—Ag2—P3	−171.6 (13)	C26—P3—C19—P2	82.8 (3)
O1—Ag1—Ag2—O1^i^	−82.88 (11)	C20—P3—C19—P2	−168.3 (2)
P2—Ag1—Ag2—O1^i^	128.03 (8)	Ag2—P3—C19—P2	−45.0 (3)
O2^i^—Ag1—Ag2—O1^i^	0.14 (10)	C32A—P2—C19—P3	−79.4 (5)
O3—Ag1—Ag2—O1^i^	−45.4 (13)	C13—P2—C19—P3	171.6 (2)
O1—Ag1—P2—C32A	27.5 (5)	C32—P2—C19—P3	−78.7 (4)
O2^i^—Ag1—P2—C32A	178.4 (5)	Ag1—P2—C19—P3	48.2 (3)
O3—Ag1—P2—C32A	−84.0 (5)	C26—P3—C20—C21	74.9 (5)
Ag2—Ag1—P2—C32A	97.3 (5)	C19—P3—C20—C21	−33.6 (5)
O1—Ag1—P2—C13	148.0 (2)	Ag2—P3—C20—C21	−157.1 (4)
O2^i^—Ag1—P2—C13	−61.1 (2)	C26—P3—C20—C25	−104.5 (4)
O3—Ag1—P2—C13	36.6 (3)	C19—P3—C20—C25	147.0 (4)
Ag2—Ag1—P2—C13	−142.17 (16)	Ag2—P3—C20—C25	23.5 (4)
O1—Ag1—P2—C19	−94.3 (2)	C25—C20—C21—C22	0.0 (9)
O2^i^—Ag1—P2—C19	56.58 (19)	P3—C20—C21—C22	−179.4 (5)
O3—Ag1—P2—C19	154.2 (3)	C20—C21—C22—C23	−0.7 (12)
Ag2—Ag1—P2—C19	−24.53 (16)	C21—C22—C23—C24	1.2 (13)
O1—Ag1—P2—C32	29.8 (4)	C22—C23—C24—C25	−0.9 (13)
O2^i^—Ag1—P2—C32	−179.4 (3)	C21—C20—C25—C24	0.2 (9)
O3—Ag1—P2—C32	−81.7 (4)	P3—C20—C25—C24	179.6 (5)
Ag2—Ag1—P2—C32	99.5 (3)	C23—C24—C25—C20	0.3 (11)
O2—Ag2—P3—C26	−30.1 (3)	C20—P3—C26—C27	−49.7 (7)
O1^i^—Ag2—P3—C26	−176.92 (19)	C19—P3—C26—C27	57.2 (7)
Ag1—Ag2—P3—C26	−100.90 (18)	Ag2—P3—C26—C27	−176.3 (6)
O2—Ag2—P3—C20	−152.0 (2)	C20—P3—C26—C31	131.9 (5)
O1^i^—Ag2—P3—C20	61.20 (19)	C19—P3—C26—C31	−121.2 (5)
Ag1—Ag2—P3—C20	137.22 (17)	Ag2—P3—C26—C31	5.3 (5)
O2—Ag2—P3—C19	91.3 (2)	C31—C26—C27—C28	0.7 (13)
O1^i^—Ag2—P3—C19	−55.48 (19)	P3—C26—C27—C28	−177.8 (8)
Ag1—Ag2—P3—C19	20.54 (16)	C26—C27—C28—C29	1.0 (16)
O2—P1—O1—Ag1	51.1 (2)	C27—C28—C29—C30	−2.3 (15)
C7—P1—O1—Ag1	−75.0 (2)	C28—C29—C30—C31	2.0 (14)
C1—P1—O1—Ag1	174.54 (18)	C27—C26—C31—C30	−1.0 (11)
O2—P1—O1—Ag2^i^	−64.2 (2)	P3—C26—C31—C30	177.5 (6)
C7—P1—O1—Ag2^i^	169.74 (19)	C29—C30—C31—C26	−0.3 (13)
C1—P1—O1—Ag2^i^	59.3 (2)	C32A—P2—C32—C33	103 (10)
P2—Ag1—O1—P1	46.6 (3)	C13—P2—C32—C33	−161.2 (5)
O2^i^—Ag1—O1—P1	−110.4 (2)	C19—P2—C32—C33	90.7 (5)
O3—Ag1—O1—P1	162.0 (3)	Ag1—P2—C32—C33	−38.2 (6)
Ag2—Ag1—O1—P1	−24.58 (17)	C32A—P2—C32—C37	−71 (10)
P2—Ag1—O1—Ag2^i^	173.13 (7)	C13—P2—C32—C37	24.8 (6)
O2^i^—Ag1—O1—Ag2^i^	16.11 (11)	C19—P2—C32—C37	−83.3 (6)
O3—Ag1—O1—Ag2^i^	−71.5 (3)	Ag1—P2—C32—C37	147.9 (5)
Ag2—Ag1—O1—Ag2^i^	101.93 (9)	C37—C32—C33—C34	0.0
O1—P1—O2—Ag2	−50.8 (3)	P2—C32—C33—C34	−174.0 (7)
C7—P1—O2—Ag2	75.9 (2)	C32—C33—C34—C35	0.0
C1—P1—O2—Ag2	−173.76 (18)	C33—C34—C35—C36	0.0
O1—P1—O2—Ag1^i^	60.5 (2)	C34—C35—C36—C37	0.0
C7—P1—O2—Ag1^i^	−172.76 (17)	C35—C36—C37—C32	0.0
C1—P1—O2—Ag1^i^	−62.5 (2)	C33—C32—C37—C36	0.0
P3—Ag2—O2—P1	−49.5 (3)	P2—C32—C37—C36	174.0 (7)
O1^i^—Ag2—O2—P1	103.7 (2)	C13—P2—C32A—C33A	−111.7 (6)
Ag1—Ag2—O2—P1	23.58 (18)	C19—P2—C32A—C33A	141.2 (6)
P3—Ag2—O2—Ag1^i^	−169.22 (8)	C32—P2—C32A—C33A	−27 (10)
O1^i^—Ag2—O2—Ag1^i^	−16.06 (11)	Ag1—P2—C32A—C33A	13.1 (7)
Ag1—Ag2—O2—Ag1^i^	−96.17 (10)	C13—P2—C32A—C37A	72.7 (8)
O1—P1—C1—C2	−155.7 (4)	C19—P2—C32A—C37A	−34.4 (9)
O2—P1—C1—C2	−27.2 (4)	C32—P2—C32A—C37A	157 (11)
C7—P1—C1—C2	88.0 (4)	Ag1—P2—C32A—C37A	−162.5 (7)
O1—P1—C1—C6	30.2 (4)	C37A—C32A—C33A—C34A	0.0
O2—P1—C1—C6	158.7 (3)	P2—C32A—C33A—C34A	−175.9 (9)
C7—P1—C1—C6	−86.1 (4)	C32A—C33A—C34A—C35A	0.0
C6—C1—C2—C3	1.8 (7)	C33A—C34A—C35A—C36A	0.0
P1—C1—C2—C3	−172.3 (4)	C34A—C35A—C36A—C37A	0.0
C1—C2—C3—C4	0.0 (9)	C35A—C36A—C37A—C32A	0.0
C2—C3—C4—C5	−2.0 (11)	C33A—C32A—C37A—C36A	0.0
C3—C4—C5—C6	2.4 (11)	P2—C32A—C37A—C36A	175.4 (10)
C4—C5—C6—C1	−0.7 (10)	O4—S3—O3—Ag1	155.6 (8)
C2—C1—C6—C5	−1.5 (7)	O5—S3—O3—Ag1	20.7 (12)
P1—C1—C6—C5	172.7 (4)	C38—S3—O3—Ag1	−90.1 (10)
O1—P1—C7—C12	156.6 (4)	O1—Ag1—O3—S3	28.5 (10)
O2—P1—C7—C12	26.0 (5)	P2—Ag1—O3—S3	178.2 (10)
C1—P1—C7—C12	−89.0 (5)	O2^i^—Ag1—O3—S3	−53.9 (10)
O1—P1—C7—C8	−31.7 (5)	Ag2—Ag1—O3—S3	−9(2)
O2—P1—C7—C8	−162.2 (5)	O3—S3—C38—F1	−130.0 (11)
C1—P1—C7—C8	82.7 (5)	O4—S3—C38—F1	−8.5 (13)
C12—C7—C8—C9	−0.1 (11)	O5—S3—C38—F1	121.2 (10)
P1—C7—C8—C9	−172.0 (7)	O3—S3—C38—F2	−13.5 (10)
C7—C8—C9—C10	−3.4 (14)	O4—S3—C38—F2	107.9 (10)
C8—C9—C10—C11	4.4 (16)	O5—S3—C38—F2	−122.3 (9)
C9—C10—C11—C12	−1.8 (14)	O3—S3—C38—F3	105.2 (10)
C8—C7—C12—C11	2.6 (9)	O4—S3—C38—F3	−133.3 (10)
P1—C7—C12—C11	174.4 (5)	O5—S3—C38—F3	−3.6 (11)
C10—C11—C12—C7	−1.7 (11)	O3B—S3B—C38B—F2B	107.0 (12)
C32A—P2—C13—C18	−75.7 (6)	O4B—S3B—C38B—F2B	−107.8 (12)
C19—P2—C13—C18	32.6 (5)	O5B—S3B—C38B—F2B	−5.3 (11)
C32—P2—C13—C18	−79.1 (5)	O3B—S3B—C38B—F3B	−25.1 (16)
Ag1—P2—C13—C18	158.1 (4)	O4B—S3B—C38B—F3B	120.1 (15)
C32A—P2—C13—C14	103.2 (5)	O5B—S3B—C38B—F3B	−137.5 (13)
C19—P2—C13—C14	−148.5 (4)	O3B—S3B—C38B—F1B	−120.1 (13)
C32—P2—C13—C14	99.8 (5)	O4B—S3B—C38B—F1B	25.1 (14)
Ag1—P2—C13—C14	−23.1 (5)	O5B—S3B—C38B—F1B	127.6 (11)
C18—C13—C14—C15	0.3 (9)		

Symmetry codes: (i) −*x*+1, −*y*+1, −*z*+1.
